# Cardiac adaptation to hypertension in adult female Dahl salt‐sensitive rats is dependent on ovarian function, but loss of ovarian function does not predict early maladaptation

**DOI:** 10.14814/phy2.13593

**Published:** 2018-02-08

**Authors:** Stian Ludvigsen, Costantino Mancusi, Simon Kildal, Giovanni de Simone, Eva Gerdts, Kirsti Ytrehus

**Affiliations:** ^1^ Cardiovascular Research Group Department of Medical Biology UiT – The Arctic University of Norway Tromsø Norway; ^2^ Hypertension Research Center Federico II University of Naples Naples Italy; ^3^ Department of Clinical Science University of Bergen Bergen Norway

**Keywords:** Collagen, echocardiography, female, gene expression, heart hypertrophy, heart remodeling, hypertension, ovariectomy

## Abstract

Aim of study was to examine experimentally the adult female hypertensive heart in order to determine the role of ovary function in the response of the heart to salt‐dependent hypertension. Dahl salt‐sensitive rats, age 12 weeks, with/without ovariectomy were fed a standard (0.3% NaCl) or high‐salt diet (8%) for 16 weeks. Mean arterial blood pressure monitored noninvasively in conscious state increased significantly by high salt. Echocardiography was performed at baseline and endpoint. Heart function and molecular changes were evaluated at endpoint by left ventricle catheterization, by sirius red staining for collagen and by gene expression using quantitative RT‐PCR for selected genes. At endpoint, significant concentric hypertrophy was present with high salt. Increase in relative wall thickening with high salt compared to normal diet was more pronounced with intact ovaries (0.33 ± 0.02 and 0.57 ± 0.04 vs. 0.29 ± 0.00 and 0.46 ± 0.03) as was the reduction in midwall fractional shortening (20 ± 0.6 and 14 ± 2 vs. 19 ± 0.9 and 18 ± 1). Ovariectomy increased stroke volume and decreased the ratio of mitral peak velocity of early filling (*E*) to early diastolic mitral annular velocity (*E*′) (*E*/*E*' ratio) when compared to hearts from intact rats. High salt increased expression of collagen I and III genes and perivascular collagen in the heart slightly, but % interstitial collagen by sirius red staining remained unchanged in intact rats and decreased significantly by ovariectomy. Added volume load but not deterioration of function or structure characterized the nonfailing hypertensive heart of salt‐sensitive females ovariectomized at mature age when compared to corresponding intact females.

## Introduction

Sex‐dependent differences in epidemiology of hypertensive heart disease are well acknowledged (Os et al. 2004). Essential hypertension contributes significantly to cardiac disease in women (Os et al. 2004; Stanton and Dunn 2017). Yet basic understanding of the female hypertensive heart with respect to mechanistic role of female steroids is not very well documented (Blenck et al. 2016). Women have lower systolic and diastolic blood pressure (BP) than men at young and mature age (Boyton 1947; Sandberg and Ji 2012), but the difference partly disappears during aging (Os et al. 2004; Sandberg and Ji 2012). Essential hypertension is assumed to represent an interaction between genetic susceptibility and life style (Oparil et al. 2003). In humans, salt sensitivity, as an acknowledged genetic factor, seems normally distributed between individuals but also contributes significantly to observed age‐dependent average increase in blood pressure (Weinberger and Fineberg 1991; Farquhar et al. 2015).

There are several experimental rat models of hypertension and hypertensive heart disease available, both invasive and noninvasive (Doggrell and Brown 1998). Similar to humans, in at least three of these rat models (spontaneously hypertensive (SHR), the stroke prone SHR (SPSHR) and Dahl salt‐sensitive (DSS) rats) hypertension occurs in males at an earlier age than in females (Dahl et al. 1975; Cambotti et al. 1984; Graham et al. 2004). Hypertensive heart failure has been repeatedly studied in the male DSS rat treated with high salt at young age (Doi et al. 2000; Klotz et al. 2006; Seymour et al. 2006; Bodyak et al. 2007; Kato et al. 2010). Dahl et al. (1975) originally showed that castration of DSS males at young age does not change the rate of development of hypertension, whereas ovariectomy of DSS females at young age accelerated hypertension development to the extent that the BP was equal to that of age‐matched males.

The primary goal of this study was to clarify how the cardiac responses to hypertension in adult female hearts were influenced by ovary function. We therefore used female DSS rats as experimental model and tested if molecular, functional or structural remodeling were influenced by the presence or absence of ovary function. Emphasis was given to the relation between collagen in the heart, gene expression and echocardiography‐ and catheter based assessment of structure and function.

## Materials and Methods

### Animals and experimental design

DSS rats were purchased from CR (Charles River Laboratories International Inc. Kingston location K90, New York, US). At arrival, all animals were maintained on normal chow containing 0.3% NaCl until they reached the age of 12 weeks, with ovariectomy being performed at the age of 11 weeks in order to allow for recovery after the surgery before starting the experiment. At age 12 weeks, animals were divided into the following groups: Female high salt with 8% NaCl (HS, *n* = 10), age‐matched controls on a normal chow (0.3% NaCl) (NS, *n* = 6) and ovariectomized females on high salt (OVX HS, *n* = 7) with age‐matched controls (OVX NS, *n* = 6). The groups stayed on the allocated diet for 16 weeks. The higher age of our rats at the starting point, compared to other studies, was chosen to ascertain that all aspects of an adult phenotype was well established and that the cardiomyocytes had reached their adult size. Blood pressure (BP) was measured weekly by noninvasive tail cuff measurement using a Coda™ Standard (Kent Scientific Corporation, Connecticut, USA). To secure that the tail was well perfused animals were placed in at heating chamber at 35°C for 20 min, before being put into restrainers placed on a heated plate (34°C) and covered with cloth blankets. Cuffs were positioned at the base of their tail, and a cycle of four habituation measurements followed by 10 normal measurements was conducted. Rats were preadapted to the procedure. Echocardiographic examination was performed at baseline and at endpoint. Finally a LV pressure volume recording was obtained before the animal was euthanized with pentobarbital, and tissues were harvested for analysis. The study was performed according to regulations of the Norwegian Animal Welfare Act (which conform to the “European Convention for the Protection of Vertebrate Animals used for Experimental and other Scientific Purposes” (Council of Europe No 123, Strasbourg 1985)) and approved by the National Animal Research Authority (FOTS ID 6784).

### Echocardiography and left ventricle pressure‐volume recordings

Animals were anesthetized with 3% isoflurane in an induction chamber, while 1.5% was delivered as maintenance anesthesia through a nose cone. Following induction, animals were placed on a heated plate in a supine position with all four legs taped to ECG electrodes on the plate. Fur was removed from the chest area before applying contact gel preheated to 37°C. Body temperature was constantly monitored using a rectal probe, and with the aid of an adjustable heating lamp, normal body temperature (37°C) was maintained during the whole examination. Baseline (age 12 weeks) echocardiographic measurements of left ventricular (LV) function and dimensions were made according to a standard protocol, and repeated after 16 weeks of dietary intervention (end‐point). Images were acquired using a VEVO 770 with a RMV 710B 38 MHz transducer (Fujifilm Visualsonics Inc., Toronto, Canada). 2D guided M‐mode images of the left ventricle (LV) were acquired from the parasternal short‐axis view at the level of papillary muscles, and LV diastolic function was assessed from pulsed wave Doppler recordings of transmitral flow velocities and tissue Doppler recordings of mitral annulus velocity acquired from the apical four‐chamber view. Heart rate (HR) was calculated from inflow at the mitral valve. Data were analyzed offline by a blinded observer using TomTec (TomTec Imaging Systems GmbH, Unterschleissheim, Germany) following current European Society of Cardiology guidelines (Nagueh et al. 2008; Lang et al. 2015). Relative wall thickness (RWT) was calculated using the formula ((PWTd + IVSTd) divided by LVIDd) where PWTd = thickness of posterior wall at diastole, IVSTd = thickness of the interventricular septum at diastole, and LVIDd = left ventricular internal diastolic dimension. Midwall fractional shortening in % was calculated as ((LVIDd + " IVSTd + " PWTd)–(LVIDs + inner shell)) divided by (LVDId + " IVSTd + " PWTd) multiplied with 100 where LVIDs = left ventricular internal diameter at systole. The inner shell in systole was calculated as [(LVIDd + " IVSTd + " PWTd)^3^–LVIDd^3^ + LVIDs^3^]^1/3^−LVIDs upon the assumption that the myocardial inner shell does not modify its mass during systole. LV mass was calculated by a validated formula indexed for tibia length (de Simone et al. 1990).

A 0.05 mg/kg injection of buprenorphine (Temgesic, Reckitt Benckiser, UK) was administered subcutaneously for analgesia following the echocardiographic examination at week 16. Under maintained isoflurane supply a 2F microtip pressure‐volume (PV) catheter (SPR‐869, Millar Instruments Inc., Houston, TX, USA) was then inserted into the LV via the right carotid artery. The PV curve was acquired using a Powerlab and Labchart 7 (AD Instruments), and off‐line analysis was performed in Labchart. Postrecording calibration for catheter‐based LV volumes was performed using values calculated from M‐mode echocardiography.

### Blood and tissue sampling

Upon completion of the PV‐recording, the catheter was withdrawn from the ventricle, and blood was sampled with a syringe through cardiac puncture. The blood was immediately centrifuged at 4°C and plasma subsequently stored in a −80°C freezer. Immediately following collection of blood, the animal was euthanized with sodium pentobarbital 100 mg/kg administered intraperitoneally. Heart, lung, thoracic aorta, and left kidney were sampled. The tibia of the right hind limb was excised for accurate measurement of length.

### ELISA

Plasma levels of brain natriuretic factor (BNP) and creatinine were measured using commercially available ELISA kits. Frozen plasma was thawed and treated according to the respective kit manual. For BNP we used Rat BNP‐45 ELISA Kit (Cat. No. KSP‐228, Nordic BioSite AB, Täby, Sweden), and for creatinine we used Rat Cr (Creatinine) ELISA Kit (Cat. No. MBS 2503978, MyBioSource Inc., San Diego, CA, USA).

### Gene expression

The apex of the LV and the thoracic aorta were stored in RNAlater (Qiagen, Hilden, Germany). Expression analyses of genes related to heart function, apoptosis, interstitial fibrosis, angiogenesis, oxidative stress, and inflammation were performed using quantitative RT‐PCR. The following genes were selected: atrial natriuretic peptide (ANP), brain natriuretic peptide (BNP), collagen type I and III (Col 1*α*1 and Col3*α*1), tissue inhibitor of matrix metalloproteinase (TIMP‐1), fibronectin (Fn‐1), myosin heavy chain (MHC) *α* and *β*, protein kinase C (PKC)‐*α*, ‐*δ*, ‐*ε*, angiotensin II receptor type 1a (AGTR 1a), angiotensin converting enzyme (ACE), tumor necrosis factor *α* (TNF‐*α*), transforming growth factor *β*1 (TGF‐*β*1), connective tissue growth factor (CTGF), p22 phox, gp91 phox, monocyte chemoattractant protein‐1 (MCP‐1), and osteopontin (SPP1).

Samples were homogenized and lysed. Total RNA was isolated according to the RNeasyFibrous Tissue protocol (Qiagen). RNA concentration was measured spectrophotometrically (NanoDrop, Witec, Switzerland) and stored at −70°C before use.

Reverse transcription of RNA was carried out using a High‐Capacity cDNA Reverse Transcription Kit (Applied Biosystems, Foster City, CA, USA).

The qRT‐PCR was performed in an ABI PRISM 7900 HT Fast real‐time thermal cycler using the SYBR green master mix (Applied Biosystems). Primers were obtained from Eurogentec (Seraing, Belgium) and Sigma‐Aldrich (St Louis, Mo, USA). The relative expression ratio of the target gene was calculated using the 2^−ΔΔCT^ method. The expression of the target genes was normalized to the stably expressed reference genes (PPIA (Cyclophilin A) and RPL13a (Ribosomal Protein L13a) or HPRT (Hypoxanthine‐guanine phosphoribosyltransferase)) based on testing by Normfinder (Andersen et al. 2004).

### Collagen content

Formalin‐fixed transverse sections of the LV were paraffin embedded and sliced with a Leica Ultracut S (Vienna, Austria). Sirius red staining of collagen fibers was performed as previously described (Jungedera et al. 1979). Twenty digital microscopic images from each heart were analyzed for percentage tissue area occupied by extracellular sirius red positive fibers using Image J (National Institutes of Health, Bethesda, MD, USA) for quantification of staining. Perivascular collagen was not included. The amount of perivascular collagen was thereafter graded by light microscopy based on the relationship between diameter of the media and the diameter of the surrounding collagen ring of the adventitia (grade 1 = diameter of collagen‐ring‐wall < 25% in comparison to diameter of media‐wall; grade 2 = 25–50%; grade 3 = 50–75%; grade 4 = 75–100%, grade 5 = >100%), blinded to information on treatment group. The perivascular collagen is presented as the mean value of the obtained grades.

### Statistics

Data analysis was performed by means of SPSS version 22 (IBM, Armonk, New York, USA). Data presented in tables are group averages ± SEM. Data were analyzed parametrically using two‐way ANOVA testing for the effect of diet and OVX, or using independent samples *t*‐test. The Holm‐Sidak post hoc test was used for multiple comparisons of groups. Echocardiography data were analyzed using repeated measures ANOVA.

## Results

### Survival

Of the original 29 female rats, two rats were removed from the experiment before the planned end‐point, one from the HS group and one from the OVX HS group. These animals were euthanized in week 13 (age 25 weeks) and week 15 (age 37 weeks) of the experiment, respectively, due to rapid loss of body mass (> 10%) and inactivity. Data from these were not included. The remaining 27 animals never showed signs of illness or discomfort during the 16‐week long experiment.

### Blood pressure

**Figure 1:**
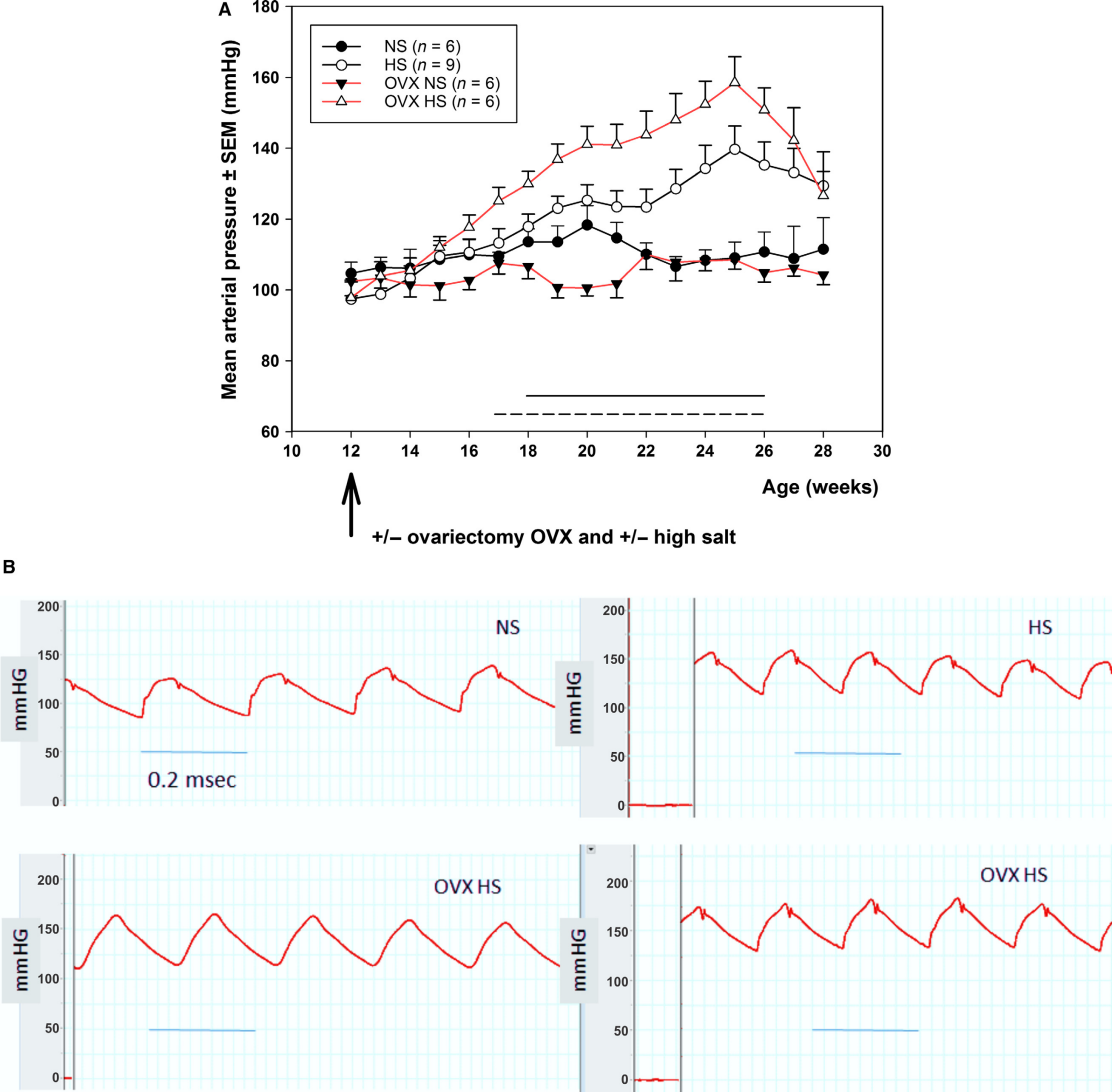
(A) Mean arterial pressure (MAP) (mean ± SEM) in intact and ovariectomized female Dahl salt‐sensitive (DSS) rats from 12 weeks of age (baseline) to 28 weeks of age (endpoint) on a normal salt diet (0.3%, NS) or a high salt diet (8%, HS). MAP was significantly higher in HS relative to NS from week 7 to 15 (indicated by solid line), and significantly higher in OVX HS relative to OVX NS from week 4 to 15 (indicated by broken line). (B) Endpoint pressure recordings from the ascending aorta of female rats anesthetized with isoflurane 1.5% plus 0.05 mg/kg buprenorphine.

The mean arterial pressure (MAP) of animals on the NS diet did not change significantly during the study (Table 1, Fig. 1A). In the two groups on HS diet, there was an increase in MAP until week 13, and thereafter MAP declined slightly (Fig. 1A). MAP in HS and OVX HS was still significantly elevated, however, when compared to the MAP at baseline (Table 1). Peak MAP was highest in OVX HS (Fig. 1A) 159 ± 18 vs. 140 ± 20 in HS, corresponding data for NS were 118 ± 3 and 109 ± 7 NS OVX. Time of peak BP occurred at different weeks for different animals. There was a significant difference between groups in MAP from age 18 to 27 weeks (Fig. 1A). Diet had a significant effect on MAP from age 21 weeks onwards, while OVX had a significant effect on MAP from age 22 to 25 weeks. The pressure measured by tail cuff at endpoint correlated with ascending aortic pressure recorded during insertion of the PV‐catheter, validating the cuff measurements (Fig. 1B).

**Table 1:** Mean arterial pressure (MAP), diastolic and systolic arterial pressure, arterial pulse pressure (PP) (mean ± SEM)

Parameter	Week	NS (*n* = 6)	HS (*n* = 9)	OVX NS (*n* = 6)	F OVX HS (*n* = 6)	Effect of diet f and/or OVX g
MAP (mmHg)	0	105 ± 3	98 ± 5	102 ± 4	98 ± 4	
	16	112 ± 9	130 ± 10^e^	104 ± 3	127 ± 7^e^	f b
Diastolic BP (mmHg)	0	94 ± 3	110 ± 5	92 ± 4	86 ± 4^c^	
	16	99 ± 10	115 ± 9^e^	96 ± 1	110 ± 17^e^	
Systolic BP (mmHg)	0	128 ± 4	122 ± 5	125 ± 7	123 ± 4	
	16	139 ± 13	162 ± 9^a^, ^d^, ^e^	137 ± 3	159 ± 6^e^	f b
PP (mmHg)	0	34 ± 1	36 ± 2	33 ± 1	37 ± 1	
	16	41 ± 4	48 ± 2^e^	41 ± 5	50 ± 2^e^	f b

a *P* < 0.05 relative to NS.

b *P* < relative to all other groups.

c *P* < relative to HS

d *P* < 0.05 relative to OVX NS.

e Elevated (*P* < 0.05) compared to week 0.

f *P* < 0.05 when analyzed according to diet.

g *P* < 0.05 when analyzed according to OVX.

### Body, organ weights, and plasma creatinine at endpoint

Average body weight (BM) in the two HS groups was significantly less compared to the NS groups after 16 weeks on HS diet (Table 2) when grouped by diet. OVX females had significantly larger BM than intact females from 6 weeks after ovariectomy to endpoint.

**Table 2:** Body mass, organ masses, blood samples, and histology at endpoint (mean ± SEM)

Parameter	NS (*n* = 6)	HS (*n* = 8–9)	OVX NS (*n* = 6)	OVX HS (*n* = 6)	Effect of diet a and/or OVX b
Body mass (g)	272 ± 6	251 ± 7	304 ± 8	278 ± 5	a b
weight gain (g)	36 ± 7.5	34 ± 6.8	65 ± 6.5	60 ± 8.3	b
LV mass (g)	0.76 ± 0.03	0.90 ± 0.05	0.82 ± 0.02	0.97 ± 0.03	a
LV mass/tibia length (g/cm)	0.18 ± 0.01	0.21 ± 0.01^c^	0.19 ± 0.01	0.22 ± 0.01^c^	a
Lung mass (g)	1.38 ± 0.04	1.76 ± 0.13	1.45 ± 0.07	1.69 ± 0.24	a
Lung mass/tibia length (g/cm)	0.33 ± 0.01	0.41 ± 0.03	0.34 ± 0.02	0.39 ± 0.05	a
Kidney mass (g)	0.85 ± 0.04	1.13 ± 0.09	0.86 ± 0.02	1.07 ± 0.04	a
Kidney mass/tibia length (g/cm)	0.20 ± 0.01	0.26 ± 0.02^c^	0.22 ± 0.01	0.25 ± 0.01	a
Plasma BNP (ng/mL)	0.30 ± 0.08	0.45 ± 0.10	0.37 ± 0.15	0.16 ± 0.03^c^	
Plasma Creatinine (*μ*g/mL)	0.13 ± 0.02	0.15 ± 0.02	0.17 ± 0.01	0.13 ± 0.03	

a *P* < 0.05 when analyzed according to diet.

b *P* < 0.05 when analyzed according to OVX.

c *P* < 0.05 relative to NS in the corresponding group.

Grouped by diet independently of OVX, females on the HS diet had significantly higher normalized LV mass than females on the NS diet, indicative of hypertrophy.

Lung and kidney mass (ww normalized to tibia length) were found to be significantly higher in the HS groups compared to the NS groups (Table 2). The increased lung wet weight and the increased kidney mass (McCormick et al. 1989), did not show interference with ovary function. Judged from measured plasma levels of creatinine there was no indication of kidney failure as the values measured were low compared to levels reported in other studies using male DSS rats (Doi et al. 2000; Klotz et al. 2006; Bodyak et al. 2007).

### Interstitial and perivascular collagen

**Figure 2:**
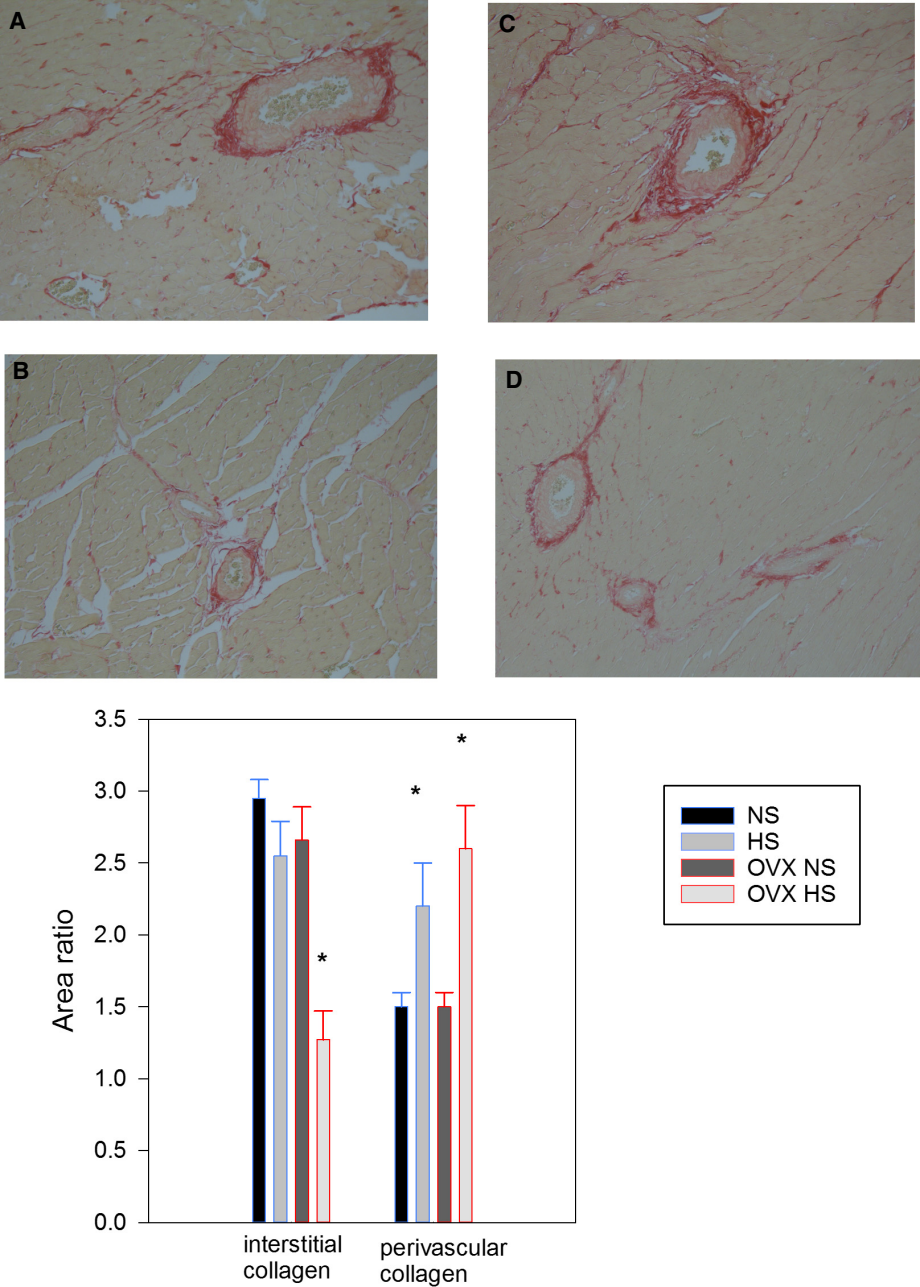
Collagen at endpoint. Top: Picro sirius red stained images of histological sections from the left ventricle of hearts harvested at endpoint. Normal salt (NS) diet (A) 200 × ) and (B) 100 × . Ovariectomized females given high salt (OVX HS) diet, (C) 200 ×  and (D) 100 × . Bottom: Semi‐quantification of interstitial and perivascular collagen based on the Sirius red staining. Normal salt NS, high salt (HS), ovariectomy with normal salt (OVX NS) and ovariectomy with HS (OVX HS) from age 12 weeks to endpoint age 28 weeks.

When analyzed for diet, the relative amount of interstitial collagen was lower in the HS groups, and perivascular collagen score was higher compared to the NS groups. Post hoc analysis showed that the F OVX HS group had significantly lower interstitial collagen compared to the other groups (Fig. 2).

### Echocardiography and LV pressure‐volumes

**Figure 3:**
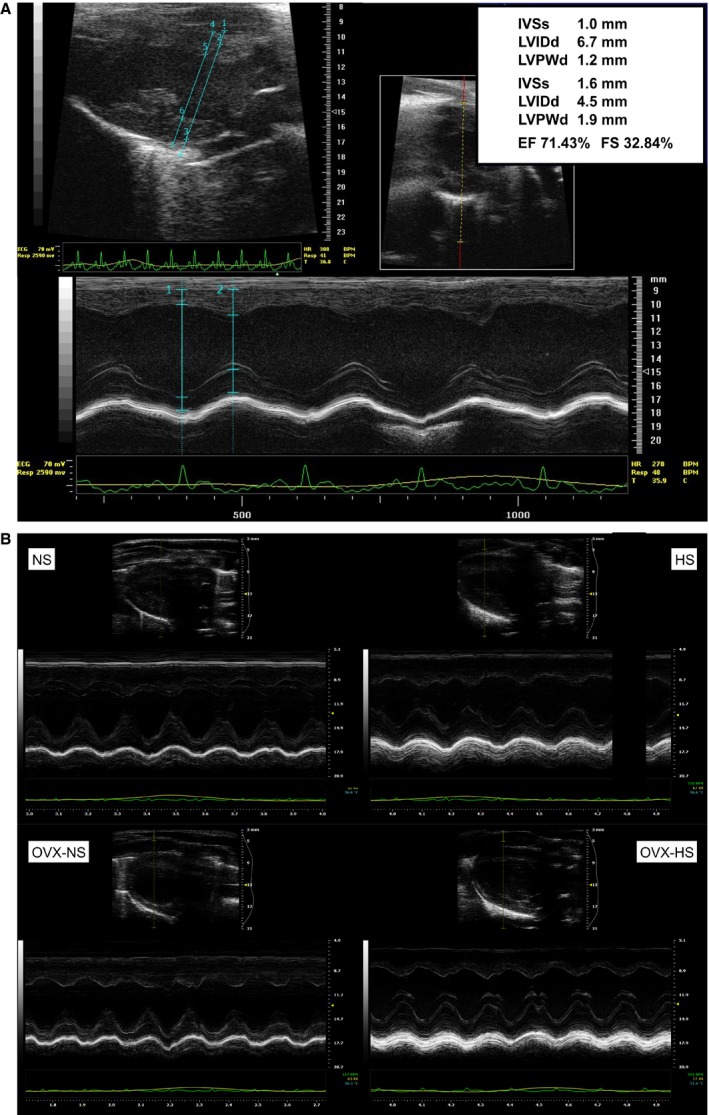
(A) Echocardiographic image (parasternal long and short axis B‐mode with corresponding M‐mode image) obtained at baseline (age 12 weeks). (B) Echocardiographic images of parasternal long axis B‐mode with corresponding M‐mode obtained from each of the four groups in the study at endpoint age 28 weeks. Normal salt NS. High salt HS. Ovariectomy and normal salt OVX NS. Ovariectomy and high salt HS OVX. Normal salt NS, high salt (HS), ovariectomy with normal salt (OVX NS) and ovariectomy with HS (OVX HS).

**Figure 4:**
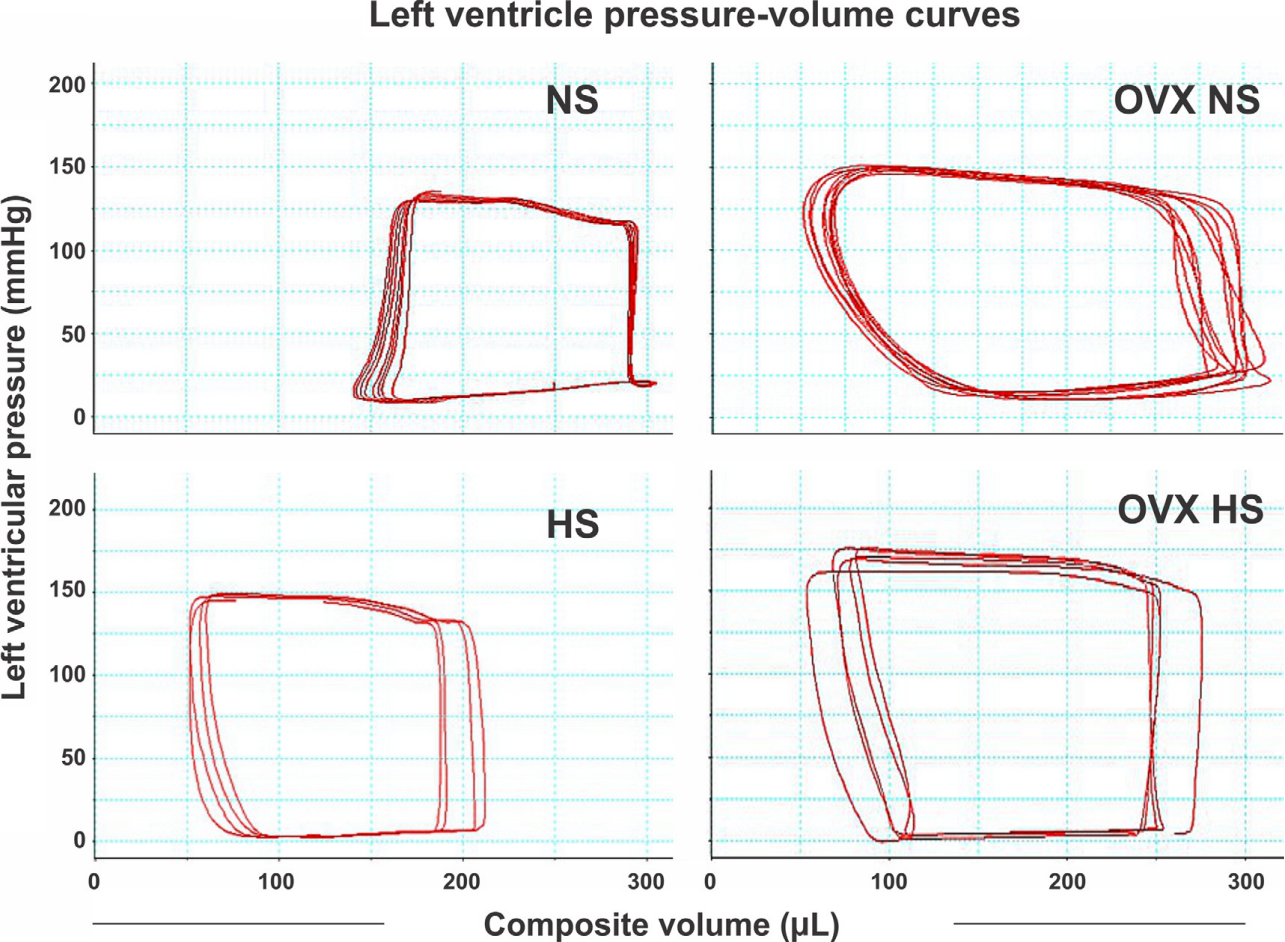
Examples of individual left ventricle pressure‐volume curves (loops) obtained at endpoint from each of the four groups in the study. Normal salt NS, high salt (HS), ovariectomy with normal salt (OVX NS) and ovariectomy with HS (OVX HS). Treatments, salt and or OVX, were from age 12 weeks to endpoint age 28 weeks.

In a subset of animals in the study, echocardiography was conducted both at baseline and at endpoint (*n* = 15) (Table 3, Fig. 3A and B), and a partly overlapping subset completed echocardiography plus LV pressure‐volume recordings at endpoint (*n* = 18) (Table 4, Fig. 4). Data on both heart geometry and heart function gathered from echocardiography at baseline were generally similar to data reported previously for both males (Watson et al. 2004) and female Sprague‐Dawley rats of similar age (Cittadini et al. 1996).

**Table 3:** Echocardiographic data on heart geometry and function at baseline (0) age 12 and endpoint (16) age 28 weeks (mean ± SEM)

Parameter		NS (*n* = 3)	HS (*n* = 5)	OVX NS (*n* = 3)	OVX HS (*n* = 4)	Effect of diet a and/or OVX b and/or time t
IVSd (mm)	0	1.0 ± 0.07	1.1 ± 0.09	1.3 ± 0.11	0.9 ± 0.13	
	16	1.2 ± 0.04	1.8 ± 0.03^d^	1.4 ± 0.19	1.7 ± 0.08	a, c
IVSs (mm)	0	1.6 ± 0.07	1.8 ± 0.25	2.1 ± 0.46	1.5 ± 0.21	
	16	2.1 ± 0.02	2.7 ± 0.13	2.3 ± 0.28	2.6 ± 0.01	c
PWd (mm)	0	1.0 ± 0.06	1.1 ± 0.10	1.2 ± 0.18	0.9 ± 0.02	
	16	1.2 ± 0.11	2.0 ± 1.28^d^	1.4 ± 0.11	1.8 ± 0.10^d^	a, c
PWs (mm)	0	1.5 ± 0.02	1.6 ± 0.17	1.8 ± 0.16	1.5 ± 0.08	
	16	1.7 ± 0.13	2.5 ± 0.06^d^	2.0 ± 0.18	2.5 ± 0.10^d^	a, c
LVIDd (mm)	0	6.7 ± 0.08	6.9 ± 0.22	7.6 ± 0.76	7.5 ± 0.24	
	16	7.4 ± 0.28	6.8 ± 0.27	8.3 ± 0.14	7.6 ± 0.14	a,b
LVIDs (mm)	0	4.2 ± 0.02	4.5 ± 0.29	4.3 ± 0.02	5.2 ± 0.25	
	16	4.7 ± 0.21	4.3 ± 0.07	5.6 ± 0.17	4.3 ± 0.15	a,b
RWT	0	0.30 ± 0.02	0.29 ± 0.02	0.31 ± 0.03	0.24 ± 0.02	
	16	0.33 ± 0.02	0.57 ± 0.04^d^	0.29 ± 0.00	0.46 ± 0.03	a, c
LVMi (g/cm)	0	0.123 ± 0.006	0.119 ± 0.008	0.129 ± 0.007	0.114 ± 0.008	
	16	0.164 ± 0.002	0.255 ± 0.012^d^	0.185 ± 0.011	0.253 ± 0.012^d^	a, c
HR (bpm)	0	338 ± 11	327 ± 14	379 ± 5	363 ± 11	b
	16	350 ± 16	363 ± 13	374 ± 37	368 ± 7	
SV (*μ*L)	0	151 ± 5	156 ± 17	161 ± 11	172 ± 12	
	16	191 ± 14	157 ± 21	223 ± 8	222 ± 11	b
CO (mL/min)	0	51 ± 27	47 ± 47	61 ± 49	62 ± 38	
	16	67 ± 38	55 ± 10	86 ± 83	82 ± 55	b
EF (%)	0	66 ± 0.6	63 ± 5	71 ± 6	57 ± 3	
	16	66 ± 0.7	65 ± 3	69 ± 2	73 ± 2^d^	a
FS (%)	0	37 ± 0.5	35 ± 4	42 ± 5	31 ± 2	
	16	37 ± 0.4	37 ± 2	33 ± 1	44 ± 2^d^	a
MFS (%)	0	20 ± 0.7	18 ± 1	20 ± 2	19 ± 0.8	
	16	20 ± 0.6	14 ± 2^d^	19 ± 0.9	18 ± 1	a
*E* to *E*′ ratio	16	15.7 ± 1.6	15.6 ± 0.6	10.0 ± 1.9	13.7 ± 1.3	b

*i* index tibia length.

a *P* < 0.05 for the effect of diet.

b *P* < 0.05 for the effect of OVX.

c *P* < 0.05 for the effect of time.

d *P* < 0.05 versus, corresponding NS.

**Table 4:** Data recorded with PV catheter presented as mean ± SEM

Parameter	NS (*n* = 6)	HS (*n* = 5)	OVX NS (*n* = 4)	OVX HS (*n* = 3)	Effect of diet and/or OVX (a)
SW (mmHg*μL)	21,018 ± 1834	21,940 ± 4077	29,288 ± 2957	30,380 ± 3196	a
CO (*μ*L/min)	63,840 ± 4595	65,090 ± 9424	84,635 ± 8040	98,200 ± 6715	a
SV (*μ*L)	194 ± 10	181 ± 27	244 ± 21	255 ± 12	a
SVi (*μ*L/g)	0.72 ± 0.05	0.74 ± 0.10	0.81 ± 0.07	0.91 ± 0.07	
LVESV (*μ*L)	78 ± 17	68 ± 4	77 ± 12	77 ± 12	
LVEDV (*μ*L)	242 ± 20	230 ± 27	286 ± 14	293 ± 10	a
Pes (mmHg)	126 ± 11	131 ± 7	133 ± 7	127 ± 11	
*P*_max_ (mmHg)	135 ± 12	145 ± 8	150 ± 7	141 ± 12	
*P*_ed_ (mmHg)	12 ± 2	16 ± 4	18 ± 4	8 ± 0.9	
*P*_min_ (mmHg)	7 ± 1	7 ± 3	7 ± 2	2 ± 0.8	
HR (bpm)	322 ± 28	359 ± 9	347 ± 18	378 ± 23	
EF (%)	77 ± 3	75 ± 3	81 ± 3	84 ± 0.2	
d*P*/d*t* max (mmHg/s)	4796 ± 472	5053 ± 291	5172 ± 179	5342 ± 535	
d*P*/d*t* min (mmHg/s)	−5096 ± 525	−5261 ± 256	−5550 ± 137	−5451 ± 602	
d*V*/d*t* max (*μ*L/s)	5303 ± 525	4930 ± 806	6221 ± 660	8334 ± 482	a
d*V*/d*t* min (*μ*L/s)	−5195 ± 629	−4236 ± 651	−5195 ± 402	−6319 ± 915	
Tau (msec)	12 ± 0.6	11 ± 0.9	11 ± 0.5	10 ± 0.2	

i index body mass.

a *P* < 0.05 when analyzed for the effect of OVX.

#### Geometry

Wall thickness (IVS and PWT), relative wall thickness (RWT), and internal chamber dimensions did not differ between groups at baseline (Table 3). A significant increase in indices of LV wall thickness and RWT was seen in HS groups at endpoint (Table 3), most evident in hearts from intact females. LV mass indexed by tibia length (LVMi) was not different between groups at baseline, but increased significantly during the intervention, and resulted in significant differences in both HS groups relative to the corresponding NS groups (Table 3). Importantly, presence or absence of ovaries during these 16 weeks did not affect LVMi despite the significant effect on body mass (Table 2).

#### Heart function

Stroke volume (SV) and cardiac output (CO) were not different between groups at baseline, but at end of the intervention OVX females had significantly higher SV and CO than intact females (Tables 3 and 4, Fig. 4). Both stoke work determined by pressure‐ volume loops and left ventricular end diastolic volume were increased at endpoint in OVX hearts. When stroke volume was indexed to body mass, the difference was no longer significant. After 16 weeks of HS diet the EF as well as FS were slightly but significantly higher in the OVX HS groups compared to corresponding NS group (Table 3). Midwall fractional shortening (MFS) was significantly reduced in the intact HS groups compared to the intact NS groups after 16 weeks of intervention (Table 3). Transmitral early velocity (E) and early mitral annulus velocity (E‘) were examined at endpoint, yielding a significantly lower E/E‘ ratio at this time for OVX females (Table 3). Isovolumetric relaxation constant (Tau) was comparable between groups (Table 4).

### Gene expression

Data are presented in Tables 5 and 6. To facilitate the comparison between groups in mRNA the expression level for each gene was normalized against the NS group (value adjusted to 1). When analyzed for the effect of OVX, *α*MHC mRNA expression levels were significantly downregulated in OVX females compared to intact females, while expression levels of *β*MHC mRNA was significantly upregulated compared to intact females (Tables 5 and 6) indicating the expected isoenzyme pattern consistent with loss of ovary function (Hydock et al. 2007).

**Table 5:** Gene expression in the apical myocardium of female Dahl SS rats normalized to housekeeping genes cyclo+RPL13a, presented as change from the mean of NS group values. The first column is official gene symbols according to HGNC

		NS (*n* = 4) Original data normalized to housekeeping genes (left). Mean set to 1 (right)		HS (*n* = 9)	OVX NS (*n* = 6)	OVX HS (*n* = 6)	Influence of diet (a) and/or OVX (b)
Heart function related genes							
NPPA	ANP	61.86 ± 25.92 × 10^−2^	1.0 ± 0.4	2.8 ± 0.7	2.2 ± 0.7	2.3 ± 0.5	
NPPB	BNP	76.24 ± 19.15 × 10^−2^	1.0 ± 0.3	1.1 ± 0.2	1.1 ± 0.2	1.1 ± 0.1	
MYH6	*α*MHC	1725 ± 154.0 × 10^−2^	1.0 ± 0.1	0.9 ± 0.1	0.7 ± 0.1	0.8 ± 0.1	b
MYH7	*β*MHC	108.54 ± 24.09 × 10^−2^	1.0 ± 0.2	1.2 ± 0.1	1.9 ± 0.2↑	2.0 ± 0.2↑	b
PRKCA	PKC*α*	1.62 ± 0.13 × 10^−2^	1.0 ± 0.1	0.9 ± 0.03	0.7 ± 0.1↓	0.8 ± 0.1↓	b
PRKCD	PKC*δ*	2.41 ± 0.36 × 10^−2^	1.0 ± 0. 2	1.1 ± 0.1	0.9 ± 0.1	0.9 ± 0.1	
PRKCE	PKC*ε*	8.88 ± 0.75 × 10^−2^	1.0 ± 0.1	0.9 ± 0.1	1.0 ± 0.1	0.9 ± 0.1	
ACE2	ACE2	0.16 ± 0.02 × 10^−2^	1.0 ± 0.2	1.0 ± 0.1	0.8 ± 0.1↓	1.0 ± 0.1	
AGTR1A	AGTR1*α*	0.18 ± 0.02 × 10^−2^	1.0 ± 0.1	1.0 ± 0.1	1.0 ± 0.04	1.1 ± 0.1	
SPP1	SPP1	1.55 ± 0.20 × 10^−2^	1.0 ± 0.1	1.7 ± 0.4	0.9 ± 0.1	1.1 ± 0.1	
Fibrosis related genes							
Col1A1	Col1*α*1	8.09 ± 1.33 × 10^−2^	1.0 ± 0.2	1.2 ± 0.1	1.0 ± 0.1	1.3 ± 0.1	a
Col3A1	Col3*α*1	26.91 ± 4.13 × 10^−2^	1.0 ± 0.2	1.4 ± 0.2	1.3 ± 0.1	1.7 ± 0.2	a
FN1	Fn‐1	0.85 ± 0.10 × 10^−2^	1.0 ± 0.1	1.3 ± 0.3	1.7 ± 0.6	1.1 ± 0.1	
TIMP1	TIMP1	1.1 ± 0.1 × 10^−2^	1.0 ± 0.1	1.8 ± 0.4	1.3 ± 0.2	1.3 ± 0.2	
TGFB1	TGF*β*1	7.52 ± 0.48 × 10^−2^	1.0 ± 0.1	1.2 ± 0.1	1.2 ± 0.1	1.1 ± 0.1	
CTGF	CTGF	0.206 ± 0.057 × 10^−2^	1.0 ± 0.3	1.4 ± 0.3	1.0 ± 0.1	1.1 ± 0.2	
Inflammation related genes							
MCP1	MCP1	0.015 ± 0.01 × 10^−2^	1.0 ± 0.4	1.6 ± 0.3	1.3 ± 0.4	0.9 ± 0.2	
TNF	TNF*α*	0.02 ± 0.01 × 10^−2^	1.0 ± 0.3	1.1 ± 0.2	1.0 ± 0.1	0.6 ± 0.1	
CYBA	CYB*α*	2.42 ± 0.35 × 10^−2^	1.0 ± 0.1	1.0 ± 0.1	1.0 ± 0.04	0.9 ± 0.1	
CYBB	CYB*β*	2.09 ± 0.50 × 10^−2^	1.0 ± 0.2	1.1 ± 0.2	1.1 ± 0.1	0.8 ± 0.1	

OVX, ovariectomized. NS, normal salt. HS, high salt. ANP, atrial natriuretic peptide; BNP, brain natriuretic pepide; MHC, myosin heavy chain; PKC, protein kinase C; TNF, tumor necrosis factor; TGF, transforming growth factor; ACE2, angiotensin converting enzyme 2; AGTR1*α*, angiotensin II receptor type 1; SPP1, secreted phosphoprotein 1 (osteopontin); Col1*α*1/3*α*1, collagen type 1 alpha 1/type 3 alpha 1; Fn‐1, fibronectin; TIMP1, tissue inhibitor of metallopeptidase 1; CTGF, connective tissue growth factor; MCP1, monocyte chemoattractant protein 1; CYB, cytochrome b. Difference from NS, ^↑^increase *P* < 0.05, ^↓^decrease *P* < 0.05.

a Significant difference when analyzed for diet.

b Significant difference when analyzed for OVX.

**Table 6:** Gene expression in the aorta of female Dahl SS rats normalized to housekeeping gene HPRT, presented as change from the NS group (mean value adjusted to 1) (first column official gene symbols according to HGNC)

		NS (*n* = 5) Left column original data normalized to housekeeping genes		HS (*n* = 6)	OVX NS (*n* = 5)	OVX HS (*n* = 8)	Influence of diet (a) and/or OVX (β)
PRKCA	PKC*α*	16.6 ± 1.5 × 10^−2^	1.0 ± 0.1	1.1 ± 0.1	1.4 ± 0.2	1.1 ± 0.1	
Col1A1	Col1*α*1	8144 ± 1830.4 × 10^−2^	1.0 ± 0.2	1.9 ± 0.2	1.3 ± 0.2	1.7 ± 0.2	a
Col3A1	Col3*α*1	2178 ± 43.8 × 10^−2^	1.0 ± 0.25	2.1 ± 0.3↑	1.2 ± 0.2	1.9 ± 0.3↑	a
MCP1	MCP1	5.8 ± 1.9 × 10^−2^	1.0 ± 0.3	1.3 ± 0.5	1.0 ± 0.3	1.0 ± 0.3	

Difference from NS, ^↑^increase *P* < 0.05, ^↓^decrease *P* < 0.05.

a *P* < 0.05 when analyzed for the effect of diet.

The Col1*α*1 and Col3*α*1 mRNA expression levels in LV as well as the thoracic aorta were upregulated in HS groups compared to the NS groups when sorted by diet (*P* < 0.05) (Tables 5 and 6). In addition, there was a significant degree of correlation between the expression level of ANF in the LV and both Col1*α*1 (*r* ^2^ = 0.708) (*P* < 0.01) and Col3*α*1 (*r* ^2^ = 0.712) (*P* < 0.01).

## Discussion

This study demonstrates that when the adult salt‐sensitive female rat develops hypertension, parallel loss of ovarian function results in changed but not impaired heart function. The hypertensive hearts of OVX female rats were characterized by increased heart work in the form of a significant increase in stroke work and volume and thereby cardiac output. Sixteen weeks with high salt supplementation resulted in concentric heart hypertrophy without any structural signs of generalized interstitial fibrosis in the left ventricle, but with slightly but significantly increased perivascular collagen deposition in conjunction with the HS diet. Opposing response to ovary status and HS seems to take place in left ventricle cavity dimensions, both systolic and diastolic. Heart function evaluated by echocardiography and pressure volume catheter indicated less reduction in midwall fractional shortening and slightly increased end‐diastolic volume in the ovariectomiced hypertensive heart.

The MCH isoform gene expression changes observed in the heart in this study are consistent with a reduction in ovary function (Hydock et al. 2007). OVX significantly increased BM compared to intact females as previously demonstrated (Hinojosa‐Laborde et al. 2000, 2004; Sasaki et al. 2000). An effect of high‐salt diet on body mass, decrease compared to controls, has previously been reported for male DSS rats (Inoko et al. 1994; Klotz et al. 2006). A corresponding decrease was not observed in a study using young (5 weeks) female DSS rats (Pfeffer et al. 1984), but in the present study of adult females we also found a trend toward reduced body weight with high‐salt diet in line with the study of male rats (Inoko et al. 1994; Klotz et al. 2006). The results of the present study are consistent with the main effect of ovarian function in salt sensitive females being limitation in volume load on the heart (preload) and thereby in heart work. Interestingly, Titze et al. (2003) was able to demonstrate reduced nonosmotic sodium storage after ovariectomy, thereby rendering these rats more prone to BP increase and volume loading during high‐salt diet consistent with the findings in the present study of an increase in MAP and stroke volume. Cardiovascular function of the DSS rat is also characterized by volume loading as well as by an afterload increase. We conclude that when ovarian function is present in the female the consequences of the volume load component are less pronounced.

In contrast to most studies of the heart of Dahl salt‐sensitive rats (males and females), we added salt in the diet and performed gonadectomy at adult age at the time when cardiomyocytes had reached adult size and female cardiovascular phenotype was well established. Age at intervention significantly modulates the response to salt‐induced elevation of blood pressure (Zicha et al. 1986, 2012). The BP response to the HS diet was more blunted in adult male animals compared to male animals starting HS diet at a younger age (Klotz et al. 2006). In a paper by Hinojosa‐Laborde et al. (2004) longitudinal BP data are presented for both intact and OVX DSS female rats which were maintained on a sodium‐deficient diet (0.1%) from age 6–7 weeks to 12 months. Even without the HS diet DSS rats would eventually develop hypertension.

Expression of genes for collagen type I and III were significantly increased by salt in both the myocardium and the aortic wall. We have previously shown significant reduction in angiotensin II‐induced collagen deposition in the pregnant heart when compared to nonpregnant state, but no difference between male and female hearts from rats exposed to higher concentrations of angiotensin II (Aljabri et al. 2011a,b) indicating that under some but not all conditions female sex hormones influence collagen deposition. We therefore tested % collagen deposition in myocardial tissue in the present study. Surprisingly we found that combined exposure to OVX and high‐salt diet resulted in a relative reduction in interstitial collagen in the left ventricle. An increase in collagen deposition characterize hypertensive male DSS rats (Inoko et al. 1994). Interstitial collagen deposition relative to cardiac growth was not increased by treatment in the female heart, but collagen tended to concentrate around vasculature, in accordance with the parallel upregulation of collagen genes in heart tissue and aorta both with and without intact ovaries. This observed reduction in percentage interstitial collagen relative to controls is probably partly a result of the combined effect of myocyte hypertrophy due to high salt and collagen remodeling due to OVX. Voloshenyuk and Gardner (2010) used volume overload by AV fistula in female rats at age 8 weeks and demonstrated similar results as in the present study with perivascular fibrosis and loss of interstitial collagen, the latter more pronounced in hearts of ovariectomized females. Lin et al. (2008) compared early and late initiation of chronic pressure overload in female rats and found loss of sirius red reactive collagen rather than an increase in collagen when salt‐dependent hypertension was introduced at older age. We therefore suggest that hormonal status as well as age could be major determinants of matrix remodeling in response to pressure and volume overload in the salt‐sensitive female heart.

The HS diet induced a gradual rise in BP as expected with the ovariectomiced group initially experiencing the largest rise in BP as previously shown (Rowland and Fregly 1992; Sasaki et al. 2000). It should be noted that we did not measure food intake in the present study and some variation in blood pressure elevation could relate to differences in salt intake due to varying appetite and physical activity during the course of the study. At endpoint, however, both HS groups demonstrated similar blood pressure levels regarding mean arterial pressure as well as arterial pulse‐pressure. In this situation, the degree of heart hypertrophy was not markedly influenced by presence or absence of ovarian function. High salt‐treated groups responded with an increase in LV mass, IVS and PW thickness and an increase in RWT reflecting concentric hypertrophy similar to what has been reported for male DSS rats (Inoko et al. 1994; Klotz et al. 2006; Bodyak et al. 2007; Louhelainen et al. 2007; Bae et al. 2011) and as expected based on results from previous work with female DSS rats (Pfeffer et al. 1984). The increase in RWT is a strong indicator of concentric geometry and a powerful determinant of reduced midwall function (Schillaci et al. 2002), which we could demonstrate as being slightly more pronounced in the intact group.

Echocardiography was performed under isoflurane influence, and supplemented with buprenorphine for catheterization of the left ventricle. Both agents might influence hemodynamic function. Compared to peripheral blood pressure in the conscious state left ventricular pressure was not significantly higher in high salt compared to the normal salt feed female rats. A possible explanation could be greater sensitivity to the hypotensive effect of isoflurane in hypertensive individuals (Yu et al. 2004).

Hypertensive heart failure was not observed in this study. There was a slight increase in lung wet‐weight with high salt, which could be due to interstitial edema. The echocardiographic examination, however, did not indicate increase in left atrial filling or elevated left ventricular diastolic pressure in the salt feed rats, which could have resulted in increased capillary pressure and fluid accumulation in the lung. A limited number of animals successfully complete all echocardiography and pressure volume recordings which compromises the power of our conclusions with respect to heart function. Difference in gene expression between the four test groups were present but minor. Accordingly, conclusion of steady state concentric hypertrophy without failure seems reasonable. Any functional effect of the MHC isoenzyme gene expression switch could not be demonstrated (Tardiff et al. 2000). This conclusion is supported by no difference between groups in values for tau (Table 4). Contractility of the heart muscle after 16 weeks treatment was not influenced by diet or ovary function. We did not find impaired active relaxation as judged from the left ventricular relaxation constant (Tau), values being comparable to those reported for healthy male rats (Xing et al. 2010). Endothelial dysfunction and diastolic dysfunction has been demonstrated in studies using intact female DSS rats treated with high salt at young age and for a duration of 12 months (Adams et al. 2015). An increase in interstitial fibrosis of the heart is one mechanism leading to deterioration in passive relaxation. Interestingly, such an increase was clearly not induced by loss of ovary function during the 16 weeks of the study.

In conclusion, hypertensive OVX Dahl salt sensitive females showed a circulatory response with increase in heart work combined with tendency to better‐maintained midwall fractional shortening in spite similar degree of compensatory LV hypertrophy and perivascular fibrosis when compared with intact hypertensive females. This might be an energy demanding situation and strengthens the need for better understanding of ovarian function in cardiac adaption to salt‐dependent hypertension in the female heart.
